# Wishful Thinking? Inside the Black Box of Exposure Assessment

**DOI:** 10.1093/annhyg/mev098

**Published:** 2016-01-13

**Authors:** Annemarie Money, Christine Robinson, Raymond Agius, Frank de Vocht

**Affiliations:** 1.Centre for Occupational and Environmental Health, Institute of Population Health, Manchester Academic Health Sciences Centre, Centre for Epidemiology, The University of Manchester, Manchester M13 9PL, UK;; 2.School of Social and Community Medicine, University of Bristol, Bristol BS8 2PS, UK

**Keywords:** exposure assessment, exposure assessment methodology, exposure estimation, expert assessment, hygiene assessment, qualitative methods, retrospective exposure assessment

## Abstract

**Background:**

Decision-making processes used by experts when undertaking occupational exposure assessment are relatively unknown, but it is often assumed that there is a common underlying method that experts employ. However, differences in training and experience of assessors make it unlikely that one general method for expert assessment would exist. Therefore, there are concerns about formalizing, validating, and comparing expert estimates within and between studies that are difficult, if not impossible, to characterize. Heuristics on the other hand (the processes involved in decision making) have been extensively studied. Heuristics are deployed by everyone as short-cuts to make the often complex process of decision-making simpler, quicker, and less burdensome. Experts’ assessments are often subject to various simplifying heuristics as a way to reach a decision in the absence of sufficient data. Therefore, investigating the underlying heuristics or decision-making processes involved may help to shed light on the ‘black box’ of exposure assessment.

**Methods:**

A mixed method study was conducted utilizing both a web-based exposure assessment exercise incorporating quantitative and semiqualitative elements of data collection, and qualitative semi-structured interviews with exposure assessors. Qualitative data were analyzed using thematic analysis.

**Results:**

Twenty-five experts completed the web-based exposure assessment exercise and 8 of these 25 were randomly selected to participate in the follow-up interview. Familiar key themes relating to the exposure assessment exercise emerged; ‘intensity’; ‘probability’; ‘agent’; ‘process’; and ‘duration’ of exposure. However, an important aspect of the detailed follow-up interviews revealed a lack of structure and order with which participants described their decision making. Participants mostly described some form of an iterative process, heavily relying on the anchoring and adjustment heuristic, which differed between experts.

**Conclusion:**

In spite of having undertaken comparable training (in occupational hygiene or exposure assessment), experts use different methods to assess exposure. Decision making appears to be an iterative process with heavy reliance on the key heuristic of anchoring and adjustment. Using multiple experts to assess exposure while providing some form of anchoring scenario to build from, and additional training in understanding the impact of simple heuristics on the process of decision making, is likely to produce a more methodical approach to assessment; thereby improving consistency and transparency in expert exposure assessment.

## INTRODUCTION

The decision processes employed by individual experts when doing qualitative, quantitative, or semiquantitative expert assessments to estimate occupational exposure remains relatively unknown. It is often assumed there is one common method by which all experts do their assessment as a result of their training (e.g. based on some official rules or guidelines) and that therefore ideally these can be captured in a conceptual model (Vadali *et al.*, 2009; Logan *et al.* 2011; Vadali *et al.*, 2012). However, it is more likely that if such a conceptual model can be found it will differ between individual experts because of their training, experience, familiarity with the process, and other factors. As a result, in those situations where the assessments cannot be directly compared to exposure measurement data it is difficult, if not impossible, to assess the quality and validity of these assessments or even to compare the assessments of different experts with each other. It is argued that this is of particular concern in multi-centre studies where the quality of the exposure assessment does not only depend on the ability of each local expert, but also on the feasibility of standardization of the work of the experts from the various distant settings (‘t Mannetje *et al.*, 2003). As a result, an important drawback of using experts is that it remains unknown how these experts make their decisions, while their estimates generally cannot be formalized, validated, and compared within and between studies (Kauppinen, 1996; Kromhout, 2002). Attempts have been made previously to gain insights into exposure assessment experts’ internal decision making processes using statistical (Burstyn *et al.*, 2013; Pronk *et al.*, 2012) or structured, deterministic (Cherrie *et al.*, 1996; Fritschi *et al.*, 2009) methods. However, because of the data and methodologies of analysis used these relied on the underlying assumption that experts use a similar ‘internal methodology’ to assess exposure. Indeed, although on average they can be used to reproduce expert’s assignments for very simple, binary metrics, e.g. such as shown for the use of classification trees (Wheeler *et al.*, 2013), significant discrepancies remain; especially for more elaborate metrics such as for example ordinal assessment (Wheeler *et al.*, 2013, 2015). A likely explanation is that experts do not all use the same assessment methodology, despite the underlying physical model being equal, as a result of differences in internal decision-making processes.

The study of heuristics in human decision making was first advanced by Simon (1972). Heuristics are deployed by everyone as short-cuts or to make the often complex process of decision-making simpler, quicker, and less burdensome. For example, more recognizable terminology describes heuristics as, a rule of thumb, trial and error, intuition, an educated guess, or just common sense (Shah and Oppenheimer, 2008). It is a way for the mind (of the decision-maker) to make decisions even though all the relevant information may not be available, where the problem is particularly complex, or where time is pressing (situations all too familiar to occupational hygienists).

Experts’ assessments are subject to various simplifying heuristics when making judgements (Vadali *et al.*, 2009). The best known of these heuristics are ‘representativeness’ (Grether, 1980; Agnoli, 1991), where the decision-maker relies on a factor being ‘representative’ or similar, categorizing, or stereotyping; ‘availability’ (Tversky and Kahneman, 1974; MacLeod and Campbell, 1992) whereby the decision-maker relies on the first aspect of the decision that they can recall, often resulting in an over-reliance on recent knowledge or experience; and ‘anchoring and adjustment’ (Northcraft and Neale, 1987; Epley and Gilovich, 2006) where the decision-maker takes an initial ‘anchor’ or reference point and then adjusts in response to further information acquired or given.

Heuristics are often employed unconsciously and, due to their subjective nature, can result in cognitive biases affecting the decision-making processes. Well-known biases that play a role are overconfidence (Koriat *et al.* 1980; Griffin and Tversky, 1992; Gigerenzer and Goldstein, 1996), confirmation bias, that is interpreting evidence in ways linked to existing beliefs or expectations, (Mynatt *et al.* 1977; Klayman 1995; Nickerson, 1998) and framing effect, i.e. how individuals react to a choice in different ways depending on whether it is positively or negatively presented (Levin *et al.*, 1998, 2002).

We conducted a mixed methods (using both quantitative and qualitative data) study to investigate exposure assessment as performed by experts to explore the internal decision making processes and heuristics involved. The quantitative expert agreement analyses, aimed at assessing the quality of the desktop exposure assessment exercise, have been reported previously (Robinson *et al.*, 2015). In brief, absolute agreement between expert raters was fair to good, and was overall better for intensity [Intraclass correlation coefficient (ICC)1 = 0.61] than for probability (ICC1 = 0.44) of exposure, and was better for experts than non-experts. Stratification for factors hypothesized to affect agreement did not show statistically significant differences, but consistent patterns contraintuitively indicated lowest agreement for medium levels of information compared with little or extensive information. Inclusion of a photo or video generally improved agreement between experts but not between non-experts.

This article explores the qualitative data that complimented Robinson *et al.* (2015) in order to shed light on the ‘black box’ of expert decision-making process and the heuristics involved (Cherrie *et al.*, 1996; Teschke *et al.*, 2002; Burstyn, 2011).

## METHODS

The methodology of the study has been described in detail elsewhere (Robinson *et al.*, 2015). In brief, we conducted a web-based desktop exposure assessment exercise in which a group of independent occupational hygienists and exposure assessors from a variety of countries were asked to assess the intensity and probability of exposure to an asthmagen for a series of job descriptions. We conducted the study for primarily the textile and cotton industries, but jobs were also included in baking, metal work and agriculture industries. The aims of the quantitative analyses were to assess agreement between experts assessing intensity and probability of exposure to airborne exposures and evaluate how well their performance compares to agreement of non-desktop based exercises reported in literature. In addition, agreement was compared with that of non-experts completing the same exercise, and results were further stratified to assess the impact of factors expected to affect assessments. All 48 cases (job descriptions) were generated from available transcripts of real interviews, testimonials, observations, photographic evidences, and videos covering a time period from 1940 to 2012. Thirty of the cases were derived from the cotton and textile industries in the UK and internationally to estimate exposure to cotton dust and the other 18 cases were from the baking, metal working and agricultural industries where exposure to other airborne asthmagens (e.g. wheat flour, welding fumes) also occurs. Low, medium, or high levels of information about each occupation were provided without any additional quantitative measurement results to enable exploration of basic strategies and advanced exposure assessment strategies. Because we were interested in the exposure assessment decision-making processes and not in the accuracy of the individual estimates, assessments were only compared to each other and not to a ‘gold standard’ such as exposure measurement data.

The expert participants included active occupational epidemiologists, academics, exposure assessors, industrial hygienists, and occupational hygienists identified from the researchers’ networks, British Occupational Hygiene annual conference attendance and publications (and considered a random selection from the respective professional communities). No other selection criteria were used.

In addition to the exposure assessment exercise, experts were also asked to describe in detail how they made their assessment for each job. This qualitative arm of the study included two components:

1. A free-text box that was completed together with the quantitative assessment during the web based exercise of 48 job descriptions. For each job description, participants were asked to explain their decisions in as much detail as possible, including the reasons for their decisions and the underlying thought processes involved; and

2. A follow-up semistructured telephone interview with a sample of those participants who had granted consent to be contacted for interview. Interviews were semistructured and allowed the opportunity to question participants on a number of topics including how accurately they believed the exercise captured their thinking process, their confidence with ratings made, important information missing from the job descriptions, whether they could ‘predict’ the top five determinants of exposure to emerge from the exercise, their awareness of making assumptions, and whether they ever questioned the validity of the job descriptions. The follow-up interviews allowed an opportunity to further refine, and plug any gaps in the data.

The interviews lasted ~30min and were transcribed verbatim and coded and analyzed using NVIVO software (QSR International, 2010) (See Box 1 for interview questions). Both components of the qualitative data collection (i.e. 25 and 8 participants, respectively) were coded and analyzed via thematic analysis. Thematic analysis (Boyatzis, 1998) is a widely used approach for the analysis of qualitative data that involves systematically identifying themes or patterns in order to categorize data, and which results in thematic structures that allow the researcher to identify commonalities, relationships and overarching patterns in the data. ‘Coding’ is the principal analytical tool used in thematic analysis in which researchers code ‘inductively’; i.e. whereby themes emerge from, and are grounded in, the data. An initial coding frame was developed based on a literature search, the pilot exercise (undertaken with three expert assessors not included in the final sample), and the experienced judgment of the researchers.

**BOX 1. INTERVIEW QUESTIONS**

Do you have any general comments on the assessment exercise that you’d like to make before we begin?

How accurately do you believe you captured your thinking process in the free text responses you gave?

If you were to do the assessment exercise again, would you do it differently? If so, how?

You will have noticed that some of the job descriptions had a very little amount of information, some had a medium amount of information and others had a lot of information. Can you give me an indication of how confident you felt for the ratings that you gave for each category?

What do you feel was missing from the job descriptions and would have been the most help to you?

Without yet knowing what themes/determinants we have identified through this research, can you tell me what determinants you would expect us to find?

(The interviewees were then shown (or told, depending on method of communication) the top five themes/determinants identified through the research.)

Can you put these top five themes/determinants in order of importance?

Is this the same order that you would consider these aspects when making a decision? If not, can you please tell me in what order you do think about these aspects?

How much did you rely on the information you were given or the experience and knowledge you already had, and did that change depending on the amount of information you were given?

Do you believe that you are always aware of when you are making assumptions?

Did you at any point question the validity of the descriptions/testimonies?

If a follow-on study is undertaken would you be prepared to participate again?

Do you have any further comments or questions?

Preliminary coding was discussed by the research team and any discrepancies/anomalies in the data were explored and reconciled on a regular basis. Qualitative data were subsequently coded to the framework and the framework adapted iteratively to be fully inclusive of all determinants/themes arising. The key research team members closely interrogated the data via a process of ‘constant comparison’ (Pope *et al.*, 2000), to identify the repeated themes, topics, relationships, and emerging patterns. The coding was reviewed and revised as the data collection continued, with any and all ambiguities in the coding discussed and reconciled by members of the research team (Silverman, 2009; Gale *et al.* 2013).

Results were described using an inductive, narrative analysis, instead of a deductive approach that is familiar to quantitative analyses (Lieblich *et al.*, 1998).

The study was reviewed and approved by the University of Manchester Research Ethics Committee (Project reference 13098).

## RESULTS

### Demographics

In total, 50 experts were approached to participate in the web-based assessment exercise. Of those, 25 completed, or partially completed, the exercise, and the free text responses from all 25 participating experts were used for component one (the online assessments) of the qualitative analysis. For component two, follow-up interviews, were undertaken with a subsample of the original 25 respondents. In total 8 experts were randomly selected interviewed over a three week period (May 2014); results from both parts of the qualitative data analysis are included in the forthcoming results.

### Key themes

The following section explores the key themes emerging from the qualitative data obtained from the free text responses elicited in the web-based exercise. In doing so, these findings will incorporate an element of discussion in order to contextualize the results. The key themes were ‘*Intensity*’ and ‘*Probability*’ (of exposure). Intensity was mentioned more often by the respondents than probability. This is possibly due to the generally perceived ease of making the decision on probability relative to the decision on intensity, or alternatively due to a perceived lack of importance of probability in comparison to intensity. This is illustrated from the follow up interviews

‘…*I think I would be thinking about probability first, but I would be saying a lot less about it, because, once that decision is made, that’s based on less information so in my mind it’s like, are they in an environment where this allergen occurs … everything else is kind of to me more related to the intensity.*’ (interview)

The theme ‘*Allergen—particulate, dust, fibre*’ was mostly relating to descriptive terminology. The respondents picked up on verbal signals given in the job descriptions that described the conditions and the work environment. Workers (cases) who described the ‘dustiness’ of their environment enabled assessors to form a visual idea of the workplace, which gave them clues as to the potential presence and concentration of the allergen providing the close relationship between this theme and those of probability and intensity. However, differences between experts also emerged here, and the use of this anecdotal evidence seemed to be more important to some participants than others. For example, one participant commented on how much the anecdotal information informed the assessment:

‘…*dust would settle on cups of tea… that tells me that that is a very, very dusty environment… it’s little clues like that for me, that influenced my thinking*’ (interview)

The ‘*Process—effects on exposure*’ theme encompassed references to the tasks undertaken and how those tasks may have affected the release of the allergen into the atmosphere. It could also relate to the physical/mechanical activity taking place or the stage of the process. For example,

‘*handling, perhaps cutting and sewing cotton fabrics would generate appreciable inhalable dust*’ (exposure exercise) and:

‘*much of the process is wet and so minimal dust exposure, potential for exposure early on, opening bags of flour, scooping into mixers, dusting’* (exposure exercise).

The theme, ‘*Duration of exposure*’ was initially coded as hours of work, but was adapted to include time spent on particular tasks, time spent in a certain area of work, other responsibilities taking employees away from or towards areas of more or less exposure potential, e.g. from the exposure exercise:

*‘…potential for exposure was intermittent to constant’*, and for example *‘…cotton dust exposure would be very likely but at a low frequency because not working always directly in the textile part of the mill*’.

The ‘*Year*’ was one of only four pieces of information that was given for every job description included in the web-based exercise. The year appears to have been used almost exclusively in a comparative way with respondents referring to it in conjunction with another year, or another job role; in this respect, it was used as a historical comparator.

*‘The work is occurring in 2012 when exposure controls would be expected to be better than in the past’* (exposure exercise)

*‘The date (1974) would suggest higher levels of exposure*’ (exposure exercise)

This theme is closely linked to another emerging from the data; ‘*Comparisons (other processes, jobs*)’. Both demonstrate a strong emphasis on the anchoring and adjustment heuristic when assessors are making their decisions. Respondents compare the job being assessed with those either previously experienced, or previously rated in the exercise. For example the following two quotes taken from the exposure exercise:

‘*I do not expect airborne concentrations to be as high as during carding, but will probably be higher than during weaving as the yarn will be more friable and I also think spinning is likely to generate more dust than weaving.*’

‘*Seamstress. Low dust exposure and little allergen present. Like Job 1*.’

### Level of information and confidence of assessment

The data from the semistructured interviews were analyzed to explore how the level of information provided to the participants affected their confidence in the ratings that they gave. For example, when more information was provided the participants were likely more willing to commit to an assessment of high or low exposure (intensity and/or probability) rating rather than a tentative and safer middle rating. Some interviewees claimed their confidence was affected by the level of information provided whereas others claimed it had no effect at all;

‘*I think it depended on the particular circumstances… you can have a very succinct description but it sounds like there’s not much exposure, whereas you can have a more extensive description but the description doesn’t focus on the information you really want to know or it’s not sufficient to get a clear idea. I can’t recall thinking ‘oh there’s more information here so I can feel more confident about the final decision’*. (interview)

‘*I think my confidence was probably proportionate to the amount of information you provided..*. *where there were photographs or even video available I was more confident still*.’ (interview)

### Approach to decision making

Many of the respondents could not identify a structure or an order to the decision making process, and indeed many described it as more of an intuitive and integrated act with heavy reliance on the anchoring and adjustment heuristic.

‘*I assume that things were better in the 1970s compared to the 1950s and 60s*.’ (exposure exercise)

‘…*my own style is a little more intuitive so sometimes it’s the assumptions, the assumptions are there but, you know, not necessarily fully explicitly stated.*’ (interview)

‘…*sometimes you’re rationalising things and sometimes you’re just doing it at a more sort of gut level*.’ (interview).

*‘I wouldn’t do it in that structured way, well I don’t know what other people think like, but my thought processes are very random I think, I wouldn’t, I wouldn’t embark on this and I didn’t embark on this with any sort of structured approach that I was going to take*.’ (interview)

Indeed, some of the interviewees mentioned the anchoring and adjustment heuristic by name, for example,

*‘…would be helpful to provide…anchoring to known scenarios…*’ (Interview)

while others made indirect references to it, e.g.

‘*I think if I’d read all the job descriptions and looked at some of the photographs and videos before I answered any of the questions I would probably have given different answers to some of the earlier ones*’. (Interview)

‘*the opportunity to view like jobs as a whole and being able to rank them together before finalising an estimate would be something that I would personally prefer to do*.’ (Interview)

‘…*I realised that I was constantly going back and forth between jobs. To say, OK, I put a 5 and a 3 for this previous job so we have one that seems to be a little bit the same so I have to go back and see what I put, see whether it’s the same… at first the first jobs were really easy ones very highly exposed so I thought they would be the highest exposed so I put 5 5 but then maybe 10 jobs down the line there’s one job that is more exposed than that or the reverse one is supposedly not exposed at all but then there’s another job that seems even less exposed so*…’ (Interview)

Almost all interviewees expressed the view that the process, i.e. understanding the task undertaken, was the most important factor and that all others would naturally follow from that. For example;

‘…*I think that process would be the first, would be the one determining the ‘is there exposure or not’, or potential for exposure. Then what would be the intensity and then it becomes difficult to give a priority but I think the process would be the first*’. (interview)

The exercise is therefore, one of relative exposure as explained by one of the participants: *‘…think of it as a relative ranking type of exercise and that’s, in my opinion that’s all what we can do if we have to retrospective estimate exposures, we can rank jobs relative to the other and in time as well to a certain extent although that its already much harder I think. But, yeah for me it’s more like ok I go through all of them and then I have a feel for ok that one must be the dustiest job so then I would scale and rank the others relative to the one that I think is the highest or the lowest…’* (interview)

## DISCUSSION

As a general observation of the qualitative data collection (i.e. not the interviews), many of the respondents appeared to find it challenging to articulate in detail the decision-making process they were undertaking. Some supplied long comprehensive responses, where others wrote one or two sentences. When asked during the follow-up interviews whether they had been able to accurately record their thinking process, the majority believed that they had and that they had explained themselves well. One admitted that they had found it ‘*challenging’* and equated that to being an *‘intuitive thinker’*. One respondent replied they thought they had captured their thought processes *‘pretty accurately’* and ‘*pretty well’*, although conceded later in the interview that

‘…*if I sat there and thought about it and mapped out my thought process I would probably arrive at something like that*…’ (interview)

Although the statement does not directly contradict the first statement, it does suggest that they may not have given such an accurate description of the process during the assessment exercise as they could have done.

Our analyses suggested that different assessors can use different methods to assess exposure which are consistent with characteristics of rational or intuitive thinkers. Those that leaned towards intuitive thinking especially found it difficult to express their decision making processes, and did not seem to use such a structured, hierarchical approach as is generally assumed to happen. We have no indications of any systematic differences in the actual estimates between rational and intuitive thinkers. These results further indicate that the decision making process seems to be primarily an iterative one, heavily reliant on anchoring and adjustment and relative comparisons.

In exposure assessment, quantitative studies have demonstrated that expert judgement can provide a valuable and reliable, if time consuming and expensive, method (see e.g. Benke, 1997; Rybicki *et al.*, 1998; Friesen *et al.* 2011; Wheeler *et al.*, 2013). Research has also shown that while experts may make relatively ‘good’ decisions, they do not necessarily do so in a rational or methodical way (Klein, 1999); and our data suggests that the same can be said for occupational exposure expert assessment. Hutton and Klein (1999) demonstrate that decision making can be ‘perceptual rather than conceptual’ and that when under time constraints, rather than assessing all available information and spending time analyzing and reaching a solution, experts will use heuristics to quickly reach their judgments; a recognitional rather than analytical approach

The experts participating in this study made strong references to visualizing and mental images and creating a perception of the task and the environment; something that seems especially relevant, although not unique, to occupational exposure assessment, and occupational hygiene. Also, several experts made mention of their lack of knowledge regarding the cotton and textile industries and many referred to gathering external information to supplement the information provided. This fits well with Hutton and Klein’s (1999) perceptual process and recognitional approach, but requiring additional strategies when presented with novel situations. The participants have also provided evidence of differences in exposure assessment methodology between the more rational and the more intuitive decision makers, suggesting that a ‘one-size fits all’ description of the decision making process is likely not appropriate. It may therefore not be such a fruitful research direction to try and establish some form of a general model for expert assessment in an attempt to ‘open the black box’ with the assumption that one such ‘black box’ exists. Instead, we argue that since our analyses indicate that one common expert assessment does most likely not exist, and because this results in incomparable assessments between individuals, studies, and industries, that more promising approaches should be based on transparent modeling of exposure measurements and contextual data where available (de Vocht *et al.*, 2008; Sanguanchaiyakrit *et al.*, 2014; Lee *et al.*, 2015), or alternatively that similarly transparent theoretical exposure assessment algorithms based on an exposure determinant-driven conceptual model for which weights are based on measurement data are likely to result in more useful ‘expert assessments’. The latter methodology has already resulted in several exposure assessment tools such as the advanced REACH tool (ART), Stoffenmanager, and ECETOC TRA (Riedmann *et al.*, 2015).

Several options have been put forward to suggest models for the way in which experts make decisions. Brunswick’s (1952) Social Judgement theory and Edwards’ (1961) Behavioural Decision Theory suggest the use of cues which are weighted and combined (Einhorn and Hogarth, 1981; Cropp *et al*., 2011). This explains the proclivity with which the experts preferentially refer to ‘key’ determinants when making their assessments. Gigerenzer and Goldstein (1996) build on Simon’s (1972) theory of bounded rationality, more particularly ‘satisficing’, where the optimal solution cannot be ascertained, a decision maker will accept a decision that satisfies needs.

Three heuristics were first described by Tversky and Kahneman (1974): ‘representativeness’, ‘availability’, and ‘anchoring and adjustment’. Of these, anchoring and adjustment has shown to be the most prevalent throughout this study. Occupational exposure assessment experts appear to rely heavily on this heuristic when retrospectively assessing exposures. In other words, many occupational hygienists and exposure assessors seem to have some mental picture of ‘general/average’ exposure in their heads that they will use as a default, and which will be adjusted based on additional information becoming available (i.e. ranking). However, because this ‘general/average’ exposure benchmark is likely to differ between experts as a result of, e.g. experience and familiarity, this may result in differences in the ‘starting position’ between the experts, which in turn may lead to different exposure scores even if the same decisions are made in the assessment procedure. This is a well-known problem of expert assessment and can to some extent be mitigated by inclusion of pre-study benchmarking exercises (‘t Mannetje, 2003).

Many of the experts seemed to struggle to express themselves and describe the processes they performed when making their decisions. It is argued that knowledge of heuristics, processes, biases and decision styles would improve the ability of experts to make more consistent and transparent decisions. Anderson (2013) describes a three step process of training, practice and application which has been shown to reduce biases arising when making judgments.

## STRENGTHS AND LIMITATIONS

Although our analyses indicated theoretical data saturation in that no new main themes were identified by adding additional participants and no additional type of assessor (in addition to intuitive and rationale) was identified anymore, the study sample included only 25 participants; eight of which also participated in the semistructured interviews.

Despite the benefits of using free text responses that were completed unstructured, there was always a risk that the responses would show a level of inconsistency both intra and interparticipant. Unfortunately, even participants who provided consistent and methodical responses did not split their explanations into the decisions regarding probability of exposure and the decisions on intensity of exposure. Therefore, these two decisions have to be judged as one and analyzed accordingly.

The assessment exercise did not completely relate to the way in which exposure assessors normally complete assessments. The online nature of the exercise differs from a more hands-on ‘on the scene’, (often article-based) approach. Therefore, the unusual format of the exercise may have influenced the way assessors approached the task. However, the information provided was similar to what is normally available to the assessors (if exposure measurement data are not available) and so was the idea of rating a set of occupations in an industry. The main difference was the novel addition of describing the decision process. As such, we do not believe that this has made a big difference, but in a way by being ‘observed’ assessors may have changed their normal process of assessment.

A strength of this study was that although exposure measurements were not available, all job descriptions were based on information available from other studies, transcripts and interviews with (ex-) workers, and as such described actual exposure circumstances in the respective job/time period combinations with the level of detail normally available.

All work done previously on expert judgment in exposure assessment has been quantitative. While this is valuable, the qualitative nature of this study has enabled a greater understanding of the decision processes frequently utilized by exposure assessors and affords new opportunities for further research in this area. As such, although the unstructured nature of the assessment descriptions was in a way a limitation of the study, this can also be considered as a strength of the approach. By not providing any guidelines on how the free text should be provided, assessors will have written this hopefully following their normal thought processes.

In conclusion, we succeeded in identifying the main themes of exposure assessment in this exercise as ‘intensity’, ‘probability’, ‘allergen’, ‘process’ and ‘duration’, which more generally describe ‘exposure level’, ‘exposure probability’, ‘agent’, ‘process (job or task)’, and ‘duration of exposure’; none of which should come as a surprise to exposure assessors. These themes were subsequently validated by the follow-up interviews in which ‘process’ was identified as the theme which held most weight to the majority of the interviewees. Nonetheless, although different experts often referred to the same themes, this study also indicated the different, and often unstructured or intuitive approaches different experts used to assess the same situations. This implies that rather than assuming that ‘any expert will do’, it would be advisable to use multiple experts to assess exposure (as has been suggested previously by others as well (Logan *et al.*, 2011; Fritschi *et al.*, 2012)), while also it is important to explicitly provide some form of anchoring and adjustment possibility for the assessors. This can be quantitative exposure measurements data, an *a priori* worked-through example of one of the occupations, or some other form of training (Friesen *et al.*, 2011; Vadali *et al.*, 2012). Exposure assessment can also be improved if additional investment is made in providing the assessors with visual information of the industries and occupations they are meant to assess.

Many of the participating experts appeared to have had difficulty in expressing their thought processes and detailing the decision-making steps that they were employing. Training assessors to improve their understanding of decision-making processes and heuristics would improve consistency, encourage a more methodical approach and thereby improve consistency and transparency in expert exposure assessment.

## FUNDING

This work was part of a project funded by the British Cotton Growing Association: Work People’s Collection Fund.

## CONFLICTS OF INTEREST

To the knowledge of the authors there are no direct or indirect conflicts of interest. The funder had no influence on preparing the research material, writing, reviewing or approving the submitted manuscript.
